# Impact of LKB1 status on radiation outcome in patients with stage III non-small-cell lung cancer

**DOI:** 10.1038/s41598-024-55476-w

**Published:** 2024-03-14

**Authors:** Piyada Sitthideatphaiboon, Chonnipa Nantavithya, Poonchavist Chantranuwat, Chanida Vinayanuwattikun, Virote Sriuranpong

**Affiliations:** 1 Division of Medical Oncology, Department of Medicine, Faculty of Medicine, Chulalongkorn University and the King Chulalongkorn Memorial Hospital, 1873 Henry Dunant Rd, Pathumwan, Bangkok, 10330 Thailand; 2 Division of Therapeutic Radiation and Oncology, Department of Radiology, Faculty of Medicine, Chulalongkorn University and the King Chulalongkorn Memorial Hospital, Bangkok, 10330 Thailand; 3 Department of Pathology, Faculty of Medicine, Chulalongkorn University and the King Chulalongkorn Memorial Hospital, Bangkok, 10330 Thailand

**Keywords:** Non-small cell lung cancer, Liver kinase B1, Nuclear factor erythroid 2-like 2, Locoregional recurrence, Radiotherapy, Non-small-cell lung cancer, Tumour biomarkers, Radiotherapy

## Abstract

Preclinical studies suggest that loss of LKB1 expression renders cancer cells less responsive to radiation partly through NRF2-mediated upregulation of antioxidant enzymes protecting against radiation-induced DNA damage. Here we investigated the association of an alteration in this pathway with radio-resistance in lung cancer patients. Patients with locally advanced non-small cell lung cancer (LA-NSCLC) who were treated with chemoradiotherapy (CRT) and analyzed for LKB1 expression using semiquantitative immunohistochemistry. Clinical characteristics and expression of LKB1 were analyzed for association with radiotherapy outcomes. We analyzed 74 available tumor specimens from 178 patients. After a median follow-up of 40.7 months, 2-year cumulative incidence of locoregional recurrence (LRR) in patients who had LKB1^Low^ expression was significantly higher than those with LKB1^High^ expression (68.8% vs. 31.3%, *P* = 0.0001). LKB1^Low^ expression was found significantly associated with a higher incidence of distant metastases (DM) (*P* = 0.0008), shorter disease-free survival (DFS) (*P* = 0.006), and worse overall survival (OS) (*P* = 0.02) compared to LKB1^High^ expression. Moreover, patients with LKB1^Low^ expression showed a significantly higher 2-year cumulative incidence of LRR (77.6% vs. 21%; *P* = 0.02), higher DM recurrence (*P* = 0.002), and shorter OS (*P* < 0.0001) compared with the *EGFR*-mutant group. For all patients with LKB1^Low^ who had LRR, these recurrences occurred within the field of radiation, in contrast to those with LKB1^High^ expression having both in-field, marginal, and out-of-field failures. LKB1 expression may serve as a potential biomarker for poor outcomes after receiving radiation in LA-NSCLC patients. Further studies to confirm the association and application are warranted.

## Introduction

Outcomes of treatment for patients with locally advanced non-small-cell lung cancer (LA-NSCLC) remain poor^1^. Radiotherapy with or without chemotherapy is the mainstay treatment for unresectable LA-NSCLC^2^. However, nearly a third of patients receiving treatment have a locoregional recurrence (LRR) within one year which is associated with reduced long-term survival^3^. There are limited data on biomarkers that can predict treatment response to radiotherapy. An increased understanding of the mechanism of resistance to radiation can help lead to improved patient selection for this treatment as well as energize novel strategies to improve treatment efficacy.

Reactive oxygen species (ROS) have been shown to play a critical role in cell death caused by ionizing radiation (IR)^4^. Therefore, inadequate removal of ROS when exposed to IR can lead to an increase in cellular oxidative stress, DNA damage, and ultimately cell death. In contrast, increased expression of antioxidant enzymes or the presence of free radical scavengers in tumor cells can lower intracellular ROS levels and confer a radio-resistance state^5^. Alteration of the Kelch-Like ECH-Associated Protein 1 (KEAP1)/ Nuclear Factor Erythroid 2-Like 2 (NRF2) pathway is a mechanism of radio-resistance utilized in many cancers, including lung cancer, by virtue of enhancing the expression of ROS scavengers and enzymes in detoxification pathways. (6–11) Altered KEAP1 function interferes with the activation of the NRF2 pathway leading to decreased expression of cytoprotective enzymes, such as NADPH quinone oxidoreductase 1 (NQO1)^6^. KEAP1/NRF2 mutations have also been shown to be associated with LRR after radiation in patients with NSCLC^7^.

Liver kinase B1 (LKB1), also known as serine/threonine protein kinase 11 (*STK11*), is a tumor-suppressor gene in NSCLC^8,9^. Recent research demonstrated that the KRAS/LKB1 mutant NSCLC is highly enriched with either KEAP1 mutations or bi-allelic loss, and expressed higher levels of NRF2-regulated genes^10,11^. Loss of LKB1, in part through NRF2-mediated upregulation of antioxidant enzymes, can protect against ROS-mediated damage and may lead to radio-resistance. (17) Preclinical studies have suggested that loss of LKB1 renders tumor cells less responsive to radiotherapy, but the interplay of these mutations with radio-resistance in NSCLC patients has not been well characterized (18–20).

To fill this research gap, our study investigated the association of LKB1 expression and outcomes of radiotherapy in patients with LA-NSCLC treated with definitive radiotherapy. The correlation between LKB1 and NRF2/NQO1 expressions was also examined.

## Results

### Patient characteristics of the study population

A total of 238 patients with LA-NSCLC patients between January 1, 2013, and December 31, 2017, at our institution. Of these, 178 patients who underwent definitive chemoradiotherapy (CRT) with curative intent were enrolled (Fig. S1). Baseline characteristics are summarized in Table 1. The median age of the study population at diagnosis was 64 years. The majority of the patients were male (73%) and had good Eastern Cooperative Oncology Group (ECOG) performance status (PS) 0 to 1 (89%). Nearly one-third (32%) were current smokers with 34% reporting as former smokers. Patients were diagnosed with non-squamous cell carcinoma (76%), T3 or T4 stage (79%), and regional lymph node involvement (95%). Eighty-nine percent of patients received concurrent chemotherapy with or without neoadjuvant or consolidation chemotherapy. Most of the patients received radiation doses more than or equal to 60 Gray (Gy) (82%). Of the 138 patients who received concurrent chemoradiation, 103 (74.6%) were able to complete the preplanned chemotherapy schedule. However, among the 18 patients who received sequential chemoradiation, only 2 (11.1%) patients were able to complete the preplanned chemotherapy schedule. All patients were treated prior to the availability of anti-programmed cell death-ligand 1 (PD-L1) durvalumab in Thailand, thus no patient received durvalumab. *EGFR* mutation and ALK status was evaluated in 69 (39%) patients. Of these, *EGFR* mutation and ALK positive was observed in 22 (32%) and 3 (4%), respectively.

**Table 1:** Patient baseline characteristics.

Baseline characteristics	All	EGFR	ALK	EGFR/ALK WT	LKB1
	N = 178	N = 22	N = 3	N = 44	N = 74
Age, years					
Median (IQR)	64 (57–69)	64.5 (57–71)	58 (50–74)	65 (58–69)	64 (58–68)
< 60	57 (32)	6 (27.3)	2 (66.7)	14 (31.8)	22 (29.7)
≥ 60	121 (68)	16 (72.7)	1 (33.3)	30 (68.2)	52 (70.3)
Gender, n (%)					
Male	129 (72.5)	8 (36.4)	1 (33.3)	31 (70.5)	55 (74.3)
Female	49 (27.5)	14 (63.6)	2 (66.7)	13 (29.5)	19 (25.7)
ECOG PS, n (%)^†^					
0–1	157 (89.2)	21 (95.5)	3 (100)	41 (93.2)	67 (90.5)
≥ 2	19 (10.8)	1 (4.5)	0	3 (6.8)	7 (9.5)
Unknown	2	0	0	0	0
Smoking status, n (%)					
Current/Former	110 (65.5)	3 (13.6)	0	27 (64.3)	49 (66.1)
Never	58 (34.5)	19 (86.4)	3 (100)	15 (35.7)	23 (31.9)
Unknown	10	0	0	2	2
Histology, n (%)					
Non-squamous	135 (75.8)	21 (95.5)	3 (100)	43 (97.7)	55 (74.3)
Squamous	43 (24.2)	1 (4.5)	0	1 (2.3)	19 (25.7)
Tumor stage, n (%)					
T1-2	37 (20.8)	8 (36.4)	2 (66.7)	12 (27.3)	18 (24.3)
T3-4	141 (79.2)	14 (63.6)	1 (33.3)	32 (72.7)	56 (75.7)
Lymph node status, n (%)					
N0-1	24 (13.5)	2 (9.1)	0	5 (11.4)	13 (17.6)
N2-3	154 (86.5)	20 (90.9)	3 (100)	39 (88.6)	61 (82.4)
Stage, n (%)^‡^					
IIIA	78 (43.8)	12 (54.5)	1 (33.3)	17 (38.6)	36 (48.6)
IIIB	100 (56.2)	10 (45.5)	2 (66.7)	27 (61.4)	38 (51.4)
Chemotherapy, n (%)					
Yes	156 (87.6)	21 (95.5)	3 (100)	43 (97.7)	69 (93.2)
No	22 (12.4)	1 (4.5)	0	1 (2.3)	5 (6.8)
CRT fashion, n (%)					
Sequential	18 (11.5)	1 (5)	0	6 (14)	6 (8.7)
Concurrent	138 (88.5)	20 (95)	3 (100)	37 (86)	63 (91.3)
CRT regimen, n (%)					
Platin-etoposide	33 (18.4)	4 (18.2)	0	8 (18.2)	16 (24.2)
Platin-paclitaxel	110 (61.8)	14 (63.6)	3 (100)	31 (70.5)	48 (72.7)
Platin-pemetrexed	8 (4.5)	2 (9.1)	0	4 (9.1)	2 (3)
Other	6 (3.3)	1 (4.5)	0	0	0
RT dose (Gy), n (%)					
< 60	31 (17.6)	0	1 (33.3)	7 (16.3)	2 (2.7)
≥ 60	145 (82.4)	22 (100)	2 (66.7)	36 (83.7)	72 (97.3)
Unknown	2	0	0	1	0
RT technique, n (%)					
3D-CRT	20 (11.6)	1 (5.3)	0	2 (4.8)	7 (9.7)
VMAT	116 (67.4)	16 (84.2)	3 (100)	29 (69)	47 (65.3)
IMRT	31 (18)	2 (10.5)	0	8 (19)	16 (22.2)
SABR	5 (3)	0	0	3 (7.2)	2 (2.8)
Unknown	6	3	0	2	2

ALK: Anaplastic lymphoma kinase, CRT: chemoradiotherapy, ECOG PS: Eastern Cooperative Oncology Group Performance Status, EGFR: epidermal growth factor receptor, IMRT: intensity-modulated radiotherapy, IQR: interquartile range, LKB1: Liver kinase B1, NSCLC: non-small cell lung cancer, NOS: not otherwise specified, SABR: stereotactic ablative radiotherapy, VMAT: volumetric modulated arc therapy, 3D-CRT: three-dimensional conformal radiotherapy.

^†^ECOG PS denotes the Eastern Cooperative Oncology Group (ECOG) scale of performance status (PS) (a performance status grade of 0 indicates asymptomatic, 1 restricted in strenuous activity but ambulatory and 2 ambulatory and capable of all self-care but unable to carry out any work activities).

^‡^Clinical staging was performed according to the seventh edition of the AJCC TNM staging system.

After a median time of follow-up 67.4 months (95% CI 62.5–72.4), 147 patients (82.6%) died, and 120 patients (67.4%) had recurrence after radiation. The initial disease recurrence manifested as LRR in 19 patients (16%), DM in 71 patients (59%) and both LRR and DM in 30 patients (25%). The median OS of the study population was 23.3 months (95% CI 18.8–27.8), whereas the median DFS was 11.0 months (95% CI 9.0–13.0).

### LKB1 expression and radiotherapy outcome

To investigate the association between LKB1 expression in tumor tissue and radiotherapy outcomes, we next analyzed the clinical parameters in correlation with tumor tissue expression of LKB1 by IHC on 74 available tumor specimens. The level LKB1 expression was determined by calculated H-scores with a median of 50 (range 0–200) as described in the method section. Baseline characteristics of these 74 patients are summarized in Table 2.

**Table 2:** Clinical characteristics of patients with locally advanced NSCLC according to LKB1 expression status.

Baseline characteristics	All	LKB1-Low	LKB1-High	*P* value
	N = 74	N = 26	N = 48	
Age, years				
Median (IQR)	64 (58–68)	62.5 (56–67)	65 (59–71)	0.29
< 60	22 (29.7)	10 (38.5)	12 (25)	
≥ 60	52 (70.3)	16 (61.5)	36 (75)	
Gender, n (%)				
Male	55 (74.3)	22 (84.6)	33 (68.7)	0.17
Female	19 (25.7)	4 (15.4)	15 (31.3)	
ECOG PS, n (%)^†^				
0–1	67 (90.5)	23 (88.5)	44 (91.7)	0.69
≥ 2	7 (9.5)	3 (11.5)	4 (8.3)	
Smoking status, n (%)				
Current/Former	49 (66.1)	18 (75)	31 (64.6)	0.43
Never	23 (31.9)	6 (25)	17 (35.4)	
Unknown	2	2	0	
Histology, n (%)				
Non-squamous	55 (74.3)	21 (80.8)	34 (70.8)	0.41
Squamous	19 (25.7)	5 (19.2)	14 (29.2)	
Tumor size (cm)				
Mean ± SD	5.69 ± 2.55	5.77 ± 1.98	5.65 ± 2.83	0.85
Tumor stage, n (%)				
T1-2	18 (24.3)	3 (11.5)	15 (31.3)	0.09
T3-4	56 (75.7)	23 (88.5)	33 (68.8)	
Lymph node status, n (%)				
N0-1	13 (17.6)	6 (23.1)	7 (14.6)	0.36
N2-3	61 (82.4)	20 (76.9)	41 (85.4)	
Stage, n (%)^‡^				
IIIA	36 (48.6)	13 (50)	23 (47.9)	1.00
IIIB	38 (51.4)	13 (50)	25 (52.1)	
Chemotherapy, n (%)				
Yes	69 (93.2)	23 (88.5)	46 (95.8)	0.34
No	5 (6.8)	3 (11.5)	2 (4.2)	
CRT fashion, n (%)				
Sequential	6 (8.7)	2 (8.7)	4 (8.7)	1.00
Concurrent	63 (91.3)	21 (91.3)	42 (91.3)	
CRT regimen, n (%)				
Platin-etoposide	16 (24.2)	7 (30.4)	9 (20.9)	0.43
Platin-paclitaxel	48 (72.7)	16 (69.6)	32 (74.4)	
Platin-pemetrexed	2 (3)	0	2 (4.7)	
RT dose (Gy), n (%)				
< 60	2 (2.7)	2 (7.7)	0	0.12
≥ 60	72 (97.3)	24 (92.3)	48 (100)	
RT technique, n (%)				
3D-CRT	7 (9.7)	2 (8)	5 (10.6)	0.74
VMAT	47 (65.3)	18 (72)	29 (61.7)	
IMRT	16 (22.2)	4 (16)	12 (25.5)	
SABR	2 (2.8)	1 (4)	1 (2.1)	
Unknown	2	1	1	

CRT: chemoradiotherapy, ECOG PS: Eastern Cooperative Oncology Group Performance Status, IMRT: intensity-modulated radiotherapy, IQR: interquartile range, LKB1: Liver kinase B1, NSCLC: non-small cell lung cancer, NOS: not otherwise specified, SABR: stereotactic ablative radiotherapy, VMAT: volumetric modulated arc therapy, 3D-CRT: three-dimensional conformal radiotherapy.

^†^ECOG PS denotes the Eastern Cooperative Oncology Group (ECOG) scale of performance status (PS) (a performance status grade of 0 indicates asymptomatic, 1 restricted in strenuous activity but ambulatory and 2 ambulatory and capable of all self-care but unable to carry out any work activities).

^‡^Clinical staging was performed according to the seventh edition of the AJCC TNM staging system.

Using ROC analysis, we chose an H-score cutoff value of 17.5 to distinguish high (≥ 17.5) versus low LKB1 expression (< 17.5), which yielded a sensitivity of 50%, a specificity of 73%, and AUC of 0.68 (95% CI 0.54–0.81) based on the occurrence of locoregional recurrence (shown in Fig. S2). There were no significant differences in clinicopathologic parameters identified between the LKB1-high and LKB1-low groups (Table 2).

**Figure 1:**
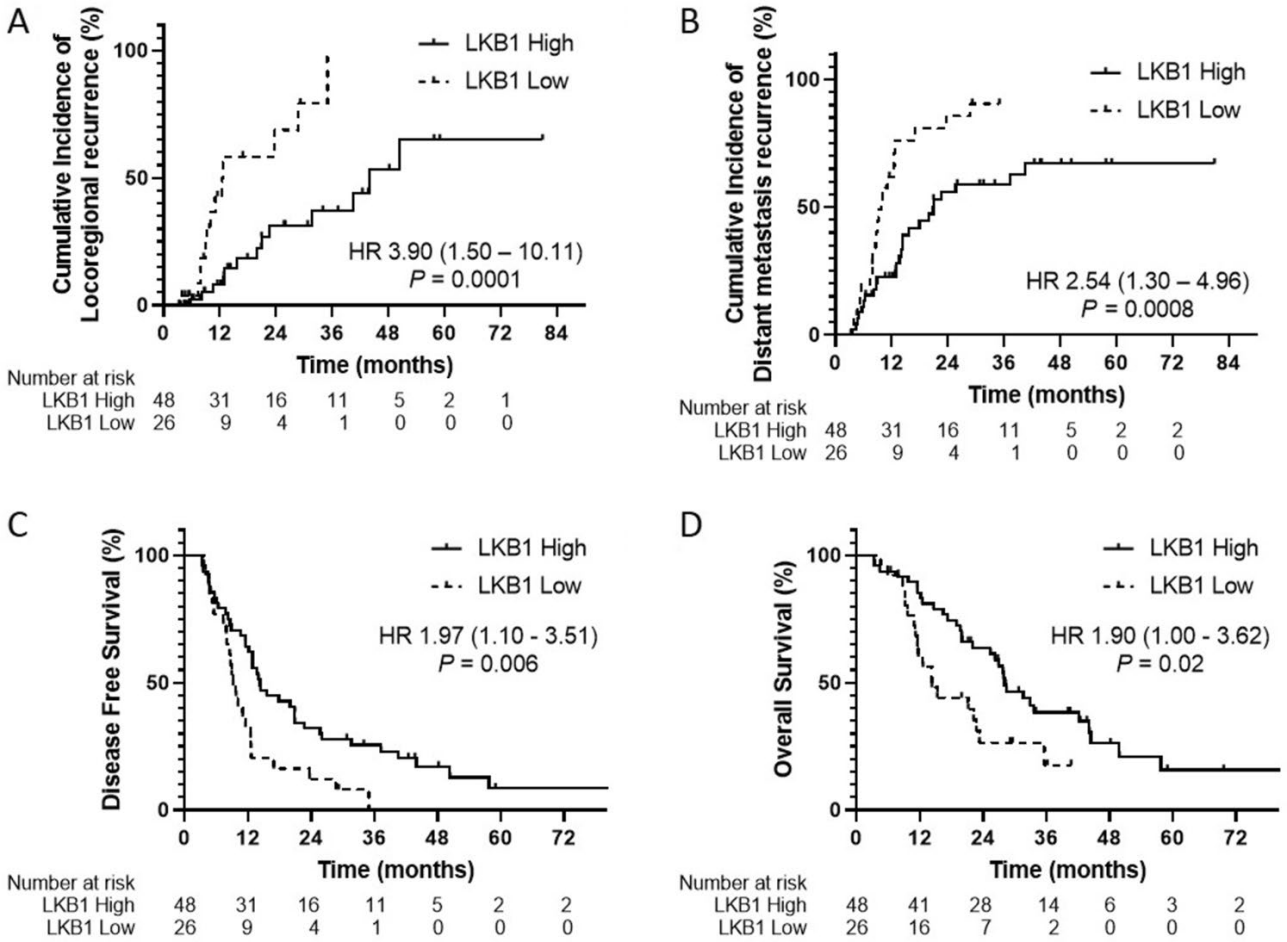
LKB1 expression status correlates with radiation outcome. (**A**) Cumulative incidence locoregional recurrence (LRR). Patients with low LKB1 expression had a significantly higher cumulative incidence of LRR than high LKB1 expression (*P* = 0.0001). (**B**) Cumulative incidence of distant metastatic (DM) recurrence. LKB1^Low^ patients was also associated with higher DM recurrence than in LKB1^High^ patients (*P* = 0.0008). (**C**) Disease-free survival (DFS). DFS was significantly worse in LKB1^Low^ patients than in LKB1^High^ patients (*P* = 0.006) and (**D**) Overall survival (OS). OS was significantly worse in LKB1^Low^ patients than in LKB1^High^ patients (*P* = 0.02).

After a median time of follow-up 40.7 months (95% CI 37.4–44.1), 51 patients (69%) died, and 53 patients (72%) had recurrence. The initial disease recurrence manifested as LRR in 7 patients (13.2%), DM in 28 patients (52.8%) and both LRR and DM in 18 patients (34%). One-year cumulative incidence of LRR and DM recurrence were 19.7% (95% CI 5.2–40.9%) and 36.6% (95% CI 22.4–50.9%), respectively. The median OS was 25.5 months (95% CI 19.2–31.7), whereas the median DFS was 12.6 months (95% CI 11.2–14.0). The 1-year cumulative incidence of LRR in patients with LKB1-low expression was 42.9% compared to 8% for LKB1-high expression. At 2 years, the cumulative incidence of LRR was 68.8% in the LKB1-low expression and 31.3% in the LKB1-high expression (HR 3.90, 95% CI 1.50–10.11, *P* = 0.0001, Fig. 1A). Low LKB1 expression was also significantly associated with higher DM recurrence (HR 2.54, 95% CI 1.30–4.96, *P* = 0.0008; Fig. 1B), shorter DFS (HR 1.97, 95% CI 1.10–3.51, *P* = 0.006; Fig. 1C), and lower OS (HR 1.90, 95% CI 1.00–3.62, *P* = 0.02; Fig. 1D) compared with LKB1-high expression. Cox regression was performed to identify additional clinicopathologic variables and LKB1 expression status correlated with DFS and OS. Multivariate analyses revealed LKB1 expression as a significant prognostic factor for both DFS (HR 2.15; 95% CI 1.21–3.80, *P* = 0.009) and OS (HR 2.03; 95% CI 1.05–3.92, *P* = 0.04) (Table 3).

**Table 3:** Univariate and multivariate analysis of DFS and OS on potential risk factors among 74 stage III NSCLC patients.

	Univariate		Multivariate	
	HR (95% CI)	*P* value	HR (95% CI)	*P* value
DFS variables†				
Age (< 60/ ≥ 60)	2.01 (1.18–3.42)	0.01*	1.93 (1.04–3.57)	0.04*
Gender (Male/ female)	1.21 (0.68–2.14)	0.52		
ECOG PS (≥ 2/ 0–1)	1.18 (0.54–2.61)	0.68	1.17 (0.51–2.70)	0.71
Smoking (never/ current-former)	1.27 (0.74–2.20)	0.39	1.10 (0.62–1.95)	0.76
Histology (Non-SQ/ SQ)	1.24 (0.69–2.22)	0.47		
T stage (T3-4/ T1-2)	0.78 (0.44–1.37)	0.38		
N stage (N2-3/ N0-1)	1.45 (0.74–2.87)	0.28		
Stage (IIIB/IIIA)	1.43 (0.87–2.34)	0.16	1.30 (0.76–2.21)	0.34
Chemotherapy (no/ yes)	1.51 (0.60–3.82)	0.38		
Radiation dose (≤ 60/ > 60)	1.38 (0.19–10.23)	0.75		
LKB1 expression (< cutoff/ ≥ cutoff)	1.97 (1.10–3.51)	0.006*	2.15 (1.21–3.80)	0.009*
OS variables†				
Age (< 60/ ≥ 60)	1.98 (1.09–3.59)	0.02*	1.73 (0.88–3.43)	0.11
Gender (Male/ female)	0.89 (0.46–1.72)	0.73		
ECOG PS (≥ 2/ 0–1)	1.24 (0.49–3.14)	0.65	1.20 (0.44–3.28)	0.72
Smoking (never/ current-former)	1.27 (0.68–2.34)	0.46	1.16 (0.59–2.30)	0.67
Histology (Non-SQ/ SQ)	1.15 (0.61–2.16)	0.67		
T stage (T3-4/ T1-2)	1.11 (0.58–2.13)	0.75		
N stage (N2-3/ N0-1)	1.68 (0.75–3.73)	0.21		
Stage (IIIB/IIIA)	1.84 (1.05–3.25)	0.04*	1.85 (1.02–3.35)	0.04*
Chemotherapy (no/ yes)	2.00 (0.79–5.10)	0.15		
Radiation dose (≤ 60/ > 60)	3.81 (0.49–29.51)	0.20		
LKB1 expression (< cutoff/ ≥ cutoff)	1.90 (1.00–3.62)	0.02*	2.03 (1.05–3.92)	0.04*

^†^Category after the slash (/) was set as reference category. CI: confidence interval, DFS: disease free survival, ECOG PS: Eastern Cooperative Oncology Group performance status, HR: hazard ratio, OS: overall survival, SQ: squamous cell carcinoma.

Significant values are in [bold].

**Figure 2:**
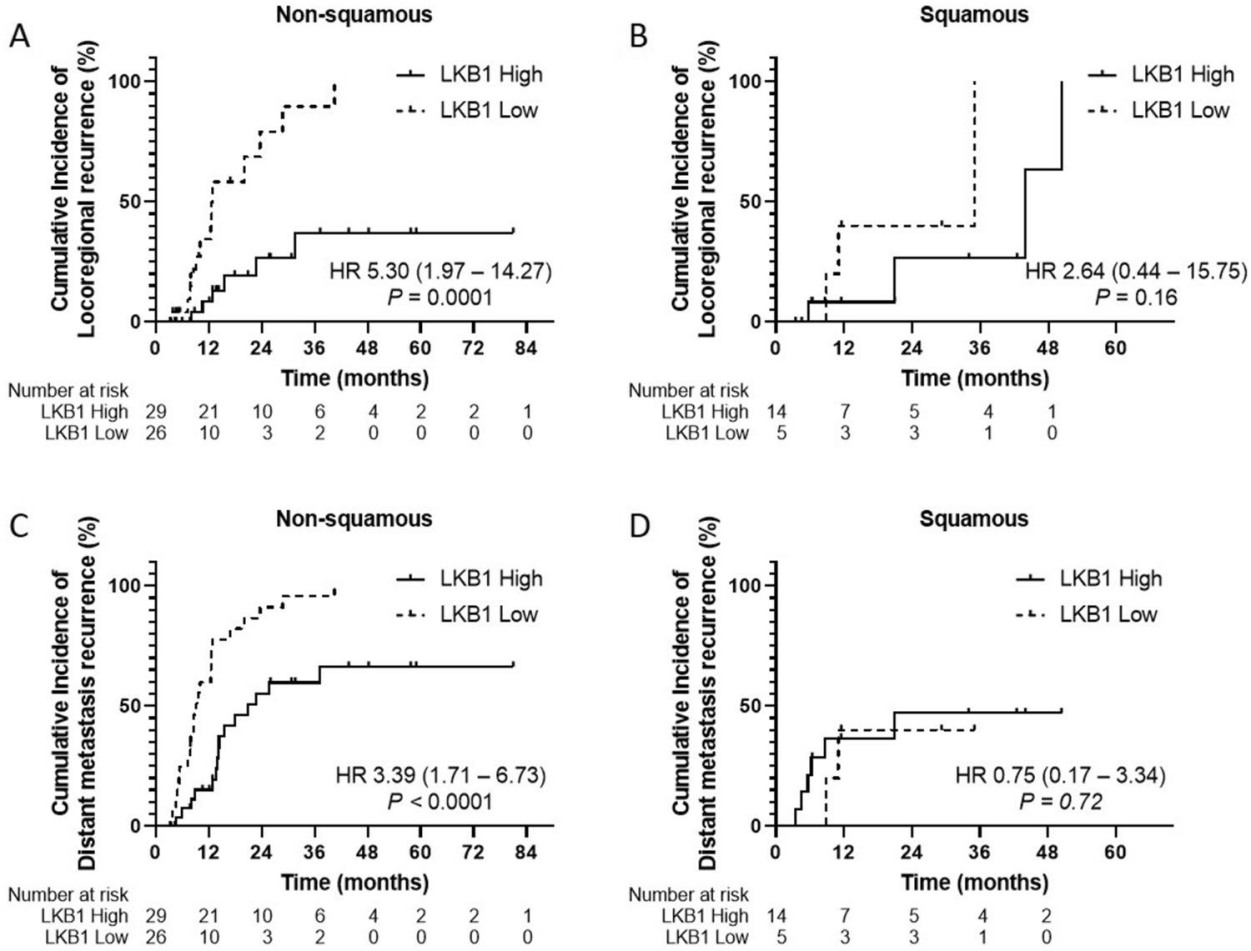
Cumulative incidence of LRR and DM recurrence according to LKB1 expression status and histologic subtype. (**A**) In non-squamous cell carcinoma subtype, LKB1^Low^ patients had a significantly higher cumulative incidence of LRR than in LKB1^High^ patients (*P* = 0.0001); however (**B**), there were not detected among squamous cell carcinoma subtype. (**C**) The cumulative incidence of DM recurrence in non-squamous cell carcinoma subtype, LKB1^Low^ patients had a significantly higher cumulative incidence of LRR than in LKB1^High^ patients (*P* < 0.0001); however (**D**), there were not detected among squamous cell carcinoma subtype.

We also found a differential effect of LKB1 expression and radiotherapy outcome by histologic subtype. In non-squamous cell carcinoma, LKB1-low expression had higher LRR (HR 5.30, 95% CI 1.97–14.27, *P* = 0.0001) and DM recurrence (HR 3.39, 95% CI 1.71–6.73, *P* < 0.0001) than LKB1-high expression (shown in Fig. 2A, C). In contrast, among squamous cell carcinoma cases, the above associations were not detected (shown in Fig. 2B, D).

### Pattern of recurrence after CRT according to EGFR mutation and LK1 status

A total of 60 patients developed relapses, including 21 patients with *EGFR* mutations and 39 patients with LKB1 group. There were no significant differences in the number of locoregional failures and distant metastasis failures between the *EGFR* mutation and LKB1 groups (Table 4). However, patients with LKB1-low expression showed significantly higher cumulative incidence of LRR (1-year was 32.7% vs. 5.9%, 2-year was 77.6% vs 21%. HR 3.42, 95% CI 0.77–15.14, *P* = 0.02). Low LKB1 expression was also significantly associated with higher DM recurrence (HR 2.58, 95% CI 1.22–5.44, *P* = 0.002), and shorted OS (HR 3.69, 95% CI 1.83–7.41, *P* < 0.0001) compared with the *EGFR*-mutant group. Moreover, LKB1-low expression group showed higher bone and adrenal gland metastasis, but less lung metastasis as compared with *EGFR*-mutant group. No significant differences were observed in LRR and DM failures between LKB1-high expression and the *EGFR*-mutant group.

**Table 4:** Pattern of failure according to *EGFR* mutation and LKB1 status.

Pattern of failure	*EGFR* mutation	LKB1-Low	LKB1-High
	N = 22	N = 17	N = 27
All failure, n (%)	21 (95.5)	17 (100)	22 (81.5)
Locoregional failure, n (%)	6 (27.3)	5 (29.4)	9 (33.3)
Primary	5 (22.7)	5 (29.4)	5 (18.5))
LN	4 (18.2)	2 (11.8)	8 (29.6)
Distant failure, n (%)	19 (86.4)	17 (100)	19 (70.4)
Lung	8 (36.4)	3 (17.6)	9 (33.3)
Pleura	2 (22.7)	5 (29.4)	3 (11.1)
Adrenal gland	1 (4.5)	4 (23.5)	3 (11.1)
Bone	4 (18.2)	6 (35.3)	3 (11.1)
Liver	2 (9.1)	2 (11.8)	0
Brain	5 (22.7)	4 (23.5)	3 (11.1)
Distant LN	1 (4.5)	1 (5.9)	1 (3.7)
Other sites	1 (4.5)	1 (5.9)	1 (3.7)

### LKB1 expression and pattern of locoregional recurrence

**Figure 3:**
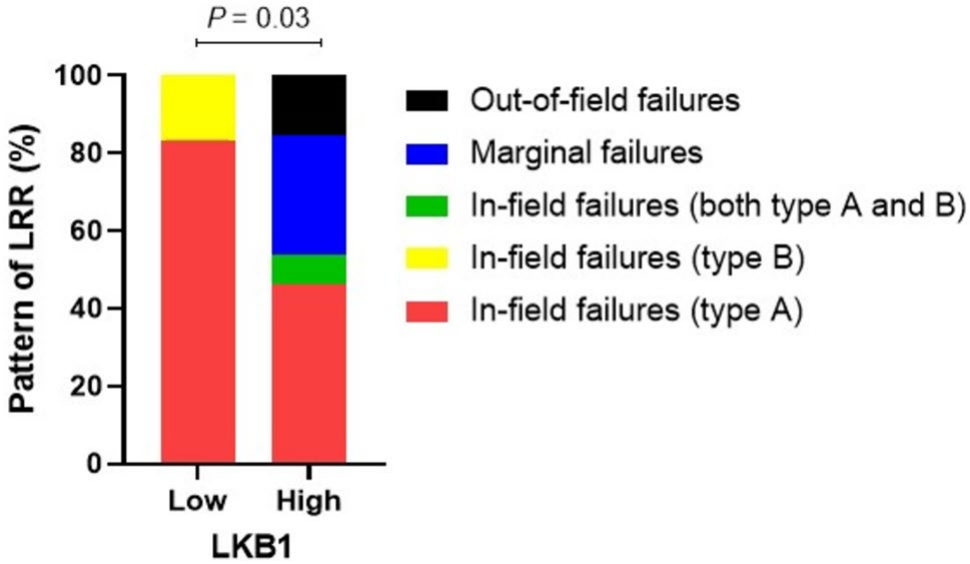
Pattern of locoregional disease according to LKB1 expression status. All LKB1^Low^ patients had in-field failures with 10 type A and 2 type B failures. LKB1^High^ patients, distribution of failure type included 7 in-field (six type A and one for both type A and B), 4 marginal and 2 out-of-field (*P* = 0.03).

To further investigate the association between LKB1 expression and radio-resistance, we performed additional analyses on the pattern of radiation failure. Among the 25 patients with LRR, 19 patients had in-field failures, 4 patients had marginal failures and 2 patients had out-of-field failures. We further classified the in-field recurrences into two groups, central high-dose, type A, and peripheral high-dose, type B. These subgroups incorporated both the location of the centroids as well as dosimetric criteria for the rGTV. There were 16 patients with type A failures, 2 patients with type B failures, and 1 patient with two separate type A and type B lesions. All patients with LKB1-low expression had in-field failures with 10 type A and 2 type B failures. Among patients with LKB1-high expression, distribution of failure type included 7 in-field (six type A and one for both type A and B), 4 marginal and 2 out-of-field (*P* = 0.03) (shown in Fig. 3). The above findings may support the influence of LKB1 expression on radiation therapy outcomes that LKB1-low expression had biological rather than technical issues underlying the majority of LRR.

### Associations between LKB1 and NRF2 expressions and its downstream target gene, NQO1

Additional IHC analyses were completed to assess whether altered expression of LKB1 was associated with the expression of downstream targets, NRF2 and NQO1. The NRF2 and NQO1 expressions were calculated with a median H-score of 50 (range 0–300) and 45 (range 0–300), respectively. We detected an inverse correlation between LKB1 expression and both NRF2 expression (r = − 0.445, *P* < 0.001) and NQO1 expression (r = − 0.302, *P* = 0.03) in the non-squamous NSCLC group. This association was not detected in patients with the squamous cell carcinoma subtype (shown in Fig. S3, S4).

## Discussion

In this study, we analyzed the outcomes of 178 LA-NSCLC patients treated with chemoradiation to determine the association with radiotherapy outcomes. A total of 67.4% of patients had disease recurrence with LRR found in 41% of those patients (49/120) with a median DFS of 11 months. These outcomes are similar to previous reports of LA-NSCLC patients receiving definitive chemoradiation^1,3^. We found a significant association between low LKB1 expression and worse outcomes, higher LRR, higher DM, shorter DFS and OS. Low LKB1 expression was found associated with a 5 times higher cumulative incidence of LRR. These findings support the role of LKB1 as a biomarker for LA-NSCLC treated with chemoradiation.

One of the most important factors influencing outcomes of LA-NSCLC patients treated with chemoradiation is the treatment intensity. Our study showed comparable radiation treatment delivery, chemotherapy regimen used, and follow-up time in both LKB1 expression groups which support the differential outcomes likely to be associated with the differential LKB1 expression rather than the treatment discrepancies. Our study also showed that all patients with LKB1-low expression who developed LRR had only in-field failures in contrast to the LKB1-high group that had both in and out-of-field failures. In addition, more type A in-field failures were observed in LKB1-low expression. These findings support the idea that radiation resistance can occur in LKB1-low expression rather than technical issues such as the planning and delivery of the radiation being the cause of radiation ineffectiveness. We also demonstrated that the association between decreased LKB1 expression and increased NRF2 expression as well as its downstream target gene, NQO1, might be the mechanism underlying the radio-resistance induced by LKB1 alteration. Finally, we found differential expressions of LKB1 depending on histological subtypes. The integrity of the LKB1/NRF2/NQO1 pathway was intact in non-squamous NSCLC, whereas this association was not observed in squamous NSCLC. We recommend further study to determine whether this pathway may be dependent on histological subtype.

Previous studies have shown that LKB1-deficient tumors are highly enriched with either KEAP1 mutations or bi-allelic loss, and express higher levels of NRF2-regulated genes as a compensatory mechanism to maintain redox homeostasis during oxidative stress. ^10,12^ More broadly, the KEAP1/NRF2 pathway has been found to be one of the mechanisms for radio-resistance in NSCLC^7,13–17^ and serves as a predictive biomarker for LRR after RT in patients with localized NSCLC^7,18^. Recent research demonstrated that LKB1-deficient tumors displayed a radio-resistant phenotype that is particularly dependent on the KEAP1/NRF2 pathway and modulation of LKB1 expression modified the sensitivity to radiation^19^. This supported our clinical observations and provided evidence for a causative role of LKB1 in modulating response to radiation.

It is noteworthy that our study analyzed results prior to the routine use of the PD-L1 inhibitor durvalumab for maintenance therapy after chemoradiotherapy, based on the phase III PACIFIC study^20,21^. Our radiation outcomes are thus not confounded by the use of the durvalumab given that LKB1/STK11 alterations may mediate resistance to PD-1/PD-L1 blockade^22^. Recent studies have suggested that both LKB1 mutations as well as the loss of LKB1 revealed an immunologically inert phenotype characterized by a markedly suppressed immune microenvironment within the tumor^10,23^. The tumor microenvironment plays a pivotal role in determining the response to radiation^24^. Therefore, an inert or “cold” tumor immune microenvironment might be contributing to radio-resistance. Further studies are needed to elucidate the role of tumor microenvironment-mediated radio-resistance mechanisms in LKB1-deficient tumors.

The primary limitation of our study is the use of retrospective data which can cause selection bias and often contain data inconsistency problems. In addition, we note that all patients were treated at a single institution which can increase data and clinical consistency but may be specific to treatment implementation at the single institution. The clinical data and pathologic samples from the patients treated with radiation according to clinical practice might affect the outcome of treatment, at least partially due to clinical selection. Furthermore, our cohort did not perform tumor genotyping, hence, we do not know if other genetic alterations are associated with radio-resistance. Moreover, the variety of genomic alterations in LKB1/STK11 and the complexities of intratumorally heterogeneity make it difficult to interpret results. Validation by other cohorts is needed to delineate the threshold of LKB1 expression that best identifies patients at increased risk of poor radiation outcomes. The complexity of LKB1 loss, which can occur through genomic and non-genomic mechanisms, can be captured by quantitative IHC for LKB1 expression and has been validated in previous studies^22,25^. Thus, the evaluation of LKB1 expression by IHC may further enhance the predictive utility of this mutation. Identification of LKB1 is a simple and cost-effective method that can be applied to clinical NSCLC specimens. Additional study is required to assess whether the role of LKB1 expression can translate into clinical practice for patients with LA-NSCLC who might be less likely to respond to radiation and more likely to suffer poor outcomes. Finally, differentiating between recurrence and post-treatment changes could be challenging, particularly after radiation therapy. The use of PET-CT can facilitate this process. However, it's crucial to note that during the acute/subacute post-treatment period, FDG avidity may also arise from inflammation. While PET/CT was not routinely utilized in most patients for radiotherapy planning and subsequent follow-up after the completion of radiation in our study, we took measures to further mitigate potential bias. The radiation oncologist and diagnostic radiologist, responsible for determining local recurrence and identifying the type of local recurrence in our study, were blinded to the results of the LKB1 status.

In summary, our study suggested that LKB1 expression may be a potential predictive marker for identifying patients with LA-NSCLC who are at risk of developing recurrence and have poor prognosis. Further validation of these findings is warranted.

## Materials and methods

### Study population

We selected retrospectively LA-NSCLC patients who received treatment at the King Chulalongkorn Memorial Hospital (KCMH) from January 2013 to December 2017. The main inclusion criteria were histologically confirmed diagnosis of NSCLC, stage III according to the 7^th^ edition of the AJCC TNM staging system who underwent definitive chemoradiotherapy (CRT) with curative intent. Archival tumor tissues were retrieved and the level of LKB1 and NRF2/NQO1 expression was determined using immunohistochemical staining (IHC). Methods indicating the study were carried out in accordance with the declarations of Helsinki. The study was approved by the Institutional Review Board (IRB) of the Faculty of Medicine, Chulalongkorn University (No.268/61). Written Informed consent was waived from individual study participants according to the ethics committee/IRB, Faculty of Medicine, Chulalongkorn University policy for retrospective study. The permission to conduct the study was approved by the director of the hospital.

### Immunohistochemistry

Formalin-fixed, paraffin-embedded (FFPE) tissue sections were prepared as per standard protocol for IHC. Epitope retrieval was performed on the Dako PT link (Dako, Denmark). Immunostaining was completed using the automated staining systems, Dako Autostainer Link48 (Dako, Denmark). The LKB1 antibody (1:100, clone D60C5F10, Cell Signaling Technology), NRF2 antibody (1:50, PA5-27,882, ThermoFisher), and NQO1 antibody (1:1000; ab28947; Abcam) were added for 30 min at room temperature. The slides were counterstained with hematoxylin. LKB1/NRF2/NQO1 expression was evaluated in the background non-neoplastic tissue providing an internal negative control. LKB1/NRF2/NQO1 staining was scored as previously described^26,27^. The staining intensity was graded as 0 (no staining), 1 + (weak), 2 + (moderate), and 3 + (intense). The percentage of stained tumor cells was recorded and the H-score was calculated using the following formula: 1 × (%cells 1 +) + 2 × (%cells 2 +) + 3 × (%cells 3 +). A final H-score of 0 was assigned as negative, 1–100 as weak, 101–200 as medium, and 201–300 + as strong. All slides were evaluated by a pathologist (P.C.) who was blinded from the patients' outcomes.

### Analysis of recurrences

Recurrence images were registered with CT simulation images that were used for radiation treatment planning. We contoured the recurrent gross tumor volume (rGTV) and the centroid (center of the rGTV) on the recurrence images and used the planning target volume (PTV) contours from the original treatment plan for further geographic analysis. Eclipse software (version 11.0.31, Varian Medical Systems, Palo Alto, USA) was used for both registration and contouring. LRR was defined as CT evidence of progressive soft tissue abnormalities or new lesions in the same lobe and/or any intrathoracic lymph node recurrence. Based on geometric data, LRR was classified as an in-field failure (centroid originating inside PTV), marginal failure (centroid originating outside PTV and recurrent lesion within 1 cm in any direction around the PTV), or out-of-field failure (centroid originating outside PTV and recurrent lesion located beyond 1 cm around the PTV). In addition, we further classified in-field recurrences into two groups using both geometric and dosimetric data. (25) Type A (central high dose) recurrences were defined as the dose to 95% of rGTV (rGTVD95%) with ≥ 95% of the dose prescribed to PTV. Type B (peripheral high dose) recurrences were defined as rGTVD95% received < 95% of the dose prescribed to PTV. Distant metastases (DM) recurrence was defined as any disease recurrence in any other location. The typical representative disease failure patterns are shown in Figure S5.

### Statistical analysis

Receiver operating characteristic (ROC) curve analysis was employed to determine the optimal cutoff value of LKB1 expression. The relationship between LKB1 expression and clinicopathological characteristics was assessed by Pearson’s chi-square test or Fisher’s exact test as appropriate. Correlations between LKB1 and NRF2/NQO1 expressions were analyzed using Spearman’s correlations. Outcomes were analyzed in terms of LRR, DM, disease-free survival (DFS), and overall survival (OS). Events (recurrence or death) were calculated from the date of diagnosis. Patients who did not develop the event at the end of the study were censored at the date of the last observation which was defined as September 22, 2019. Univariate and multivariate analyses were performed using the Cox model and hazard ratios (HRs) and 95% confidence intervals (95% CIs) were calculated. *P*-values < 0.05 were considered statistically significant. All statistical analyses were conducted using GraphPad Prism version 8.00 for Windows (GraphPad Software, La Jolla, California, USA) and SPSS 23.0 (SPSS Inc, Chicago, Illinois, USA).

### Supplementary Information

Supplementary Figures.

## Data Availability

The datasets used and/or analyzed during the current study available from the corresponding author (V.S) on reasonable request.

## Abbreviations

DFS: Disease-free survival
DM: Distant metastases
ECOG PS: Eastern cooperative oncology group performance status
IHC: Immunohistochemistry
KEAP1: Kelch-Like ECH-associated protein 1
LA-NSCLC: Locally advanced non-small-cell lung cancer
LKB1: Liver kinase B1
LRR: Locoregional recurrence
NQO1: NADPH quinone oxidoreductase 1
NRF2: Nuclear factor erythroid 2-like 2
OS: Overall survival
ROC: Receiver operating characteristic
RT: Radiotherapy
STK11: Serine/threonine protein kinase
