# Supplementary material for: Impact of LKB1 status on radiation outcome in patients with stage III non-small-cell lung cancer
Source: Sci Rep. 2024 Mar 14;14:6146. doi: 10.1038/s41598-024-55476-w (PMC10938003; doi:10.1038/s41598-024-55476-w)

**Supplementary Information**

**Supplementary Fig. S1**. Flow chart of patients enrolled in the study.

**Supplementary Fig. S2.** The cut-off value of LKB1 expression H-score with ROC curve analysis.

**Supplementary Fig. S3.** Associations between LKB1 and NRF2 expressions and its downstream target gene, NQO1, according to the histological subtypes. In non-squamous cell carcinoma subtype, relationships between the expression of LKB1 and (A) NRF2 expression, and (B) NQO1 expression. In squamous cell carcinoma subtype, relationships between the expression of LKB1 and (C) NRF2 expression, and (D) NQO1 expression. Tumor LKB1, NRF2, and NQO1 expressions are quantified as H-score.

**Supplementary Fig. S4.** Spearman’s correlation coefficient between LKB1, NRF2 and NQO1 expressions according to the histological subtypes.

**Supplementary Fig. S5.** Representative disease failure patterns: (A) type A in-field recurrence, (B) type B in-field recurrence, (C) marginal in-failure recurrence, (D) out-of-field recurrence. (E) and (F) show radiation doses registered with recurrence images of type A and type B in-field recurrence, respectively. Contouring and radiation dose. The green contour is the centroid, the magenta contour is the recurrent gross tumor volume (rGTV), and the red contour is the primary planning target volume (PTV).

Supplementary Figure 1.


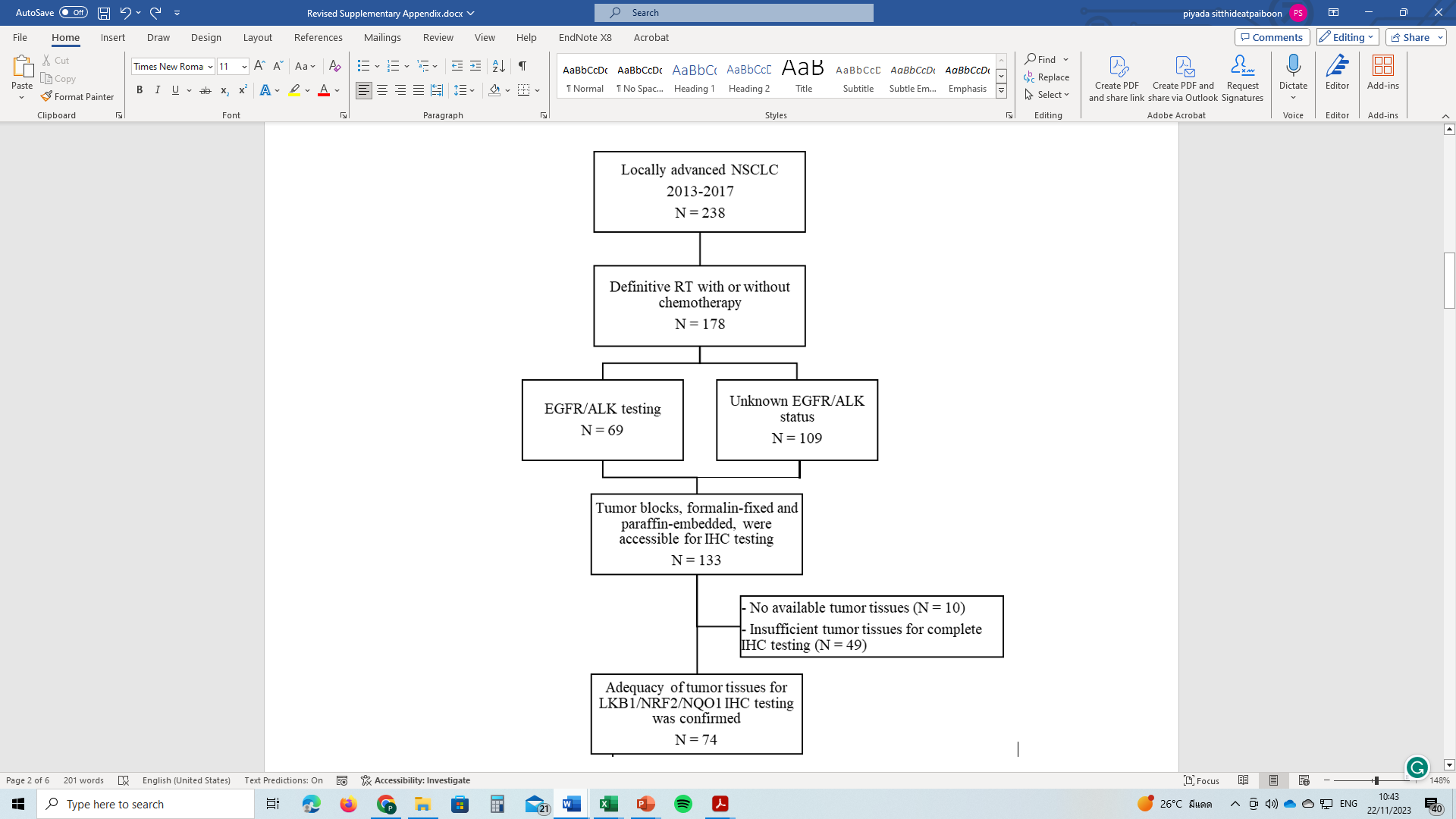


Supplementary Figure 2.


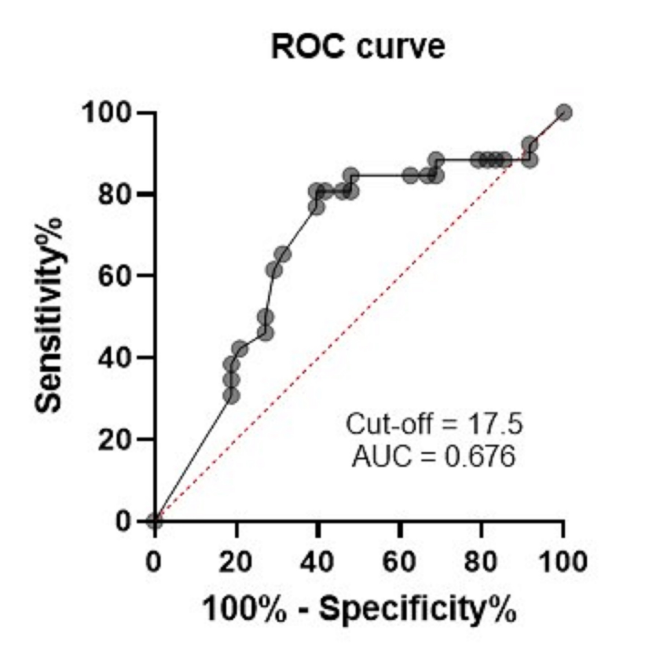


Supplementary Figure 3.


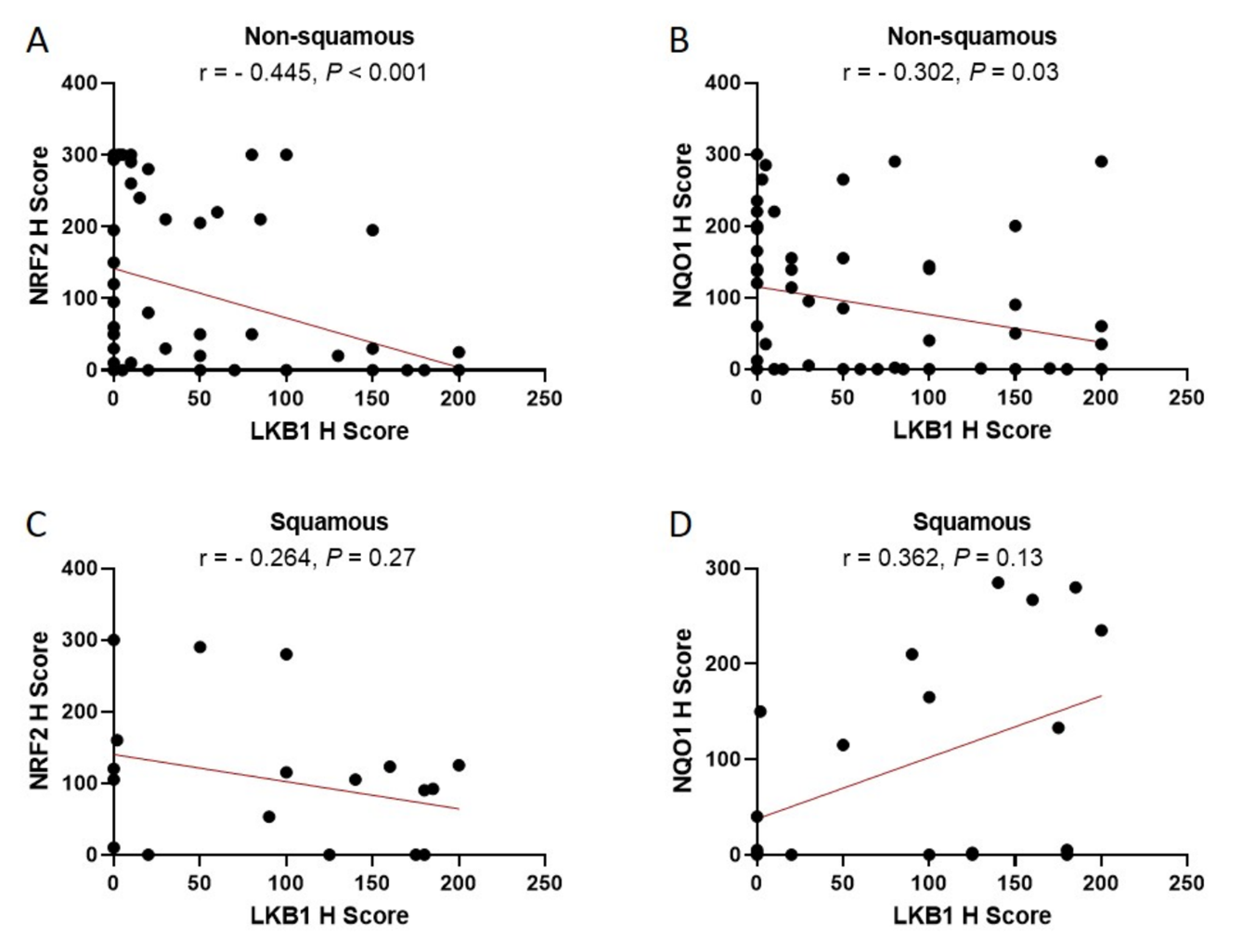


Supplementary Figure 4.


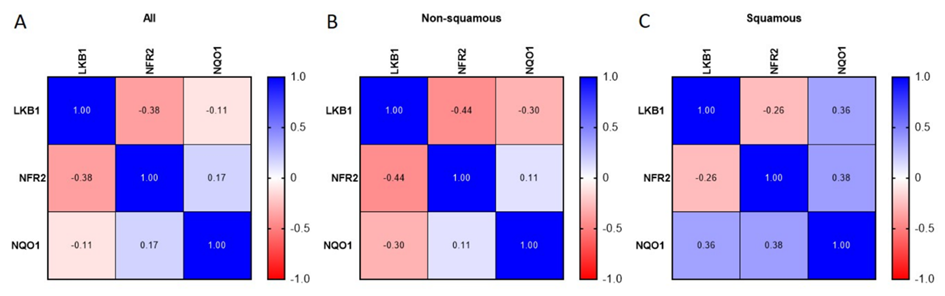


Supplementary Figure 5.


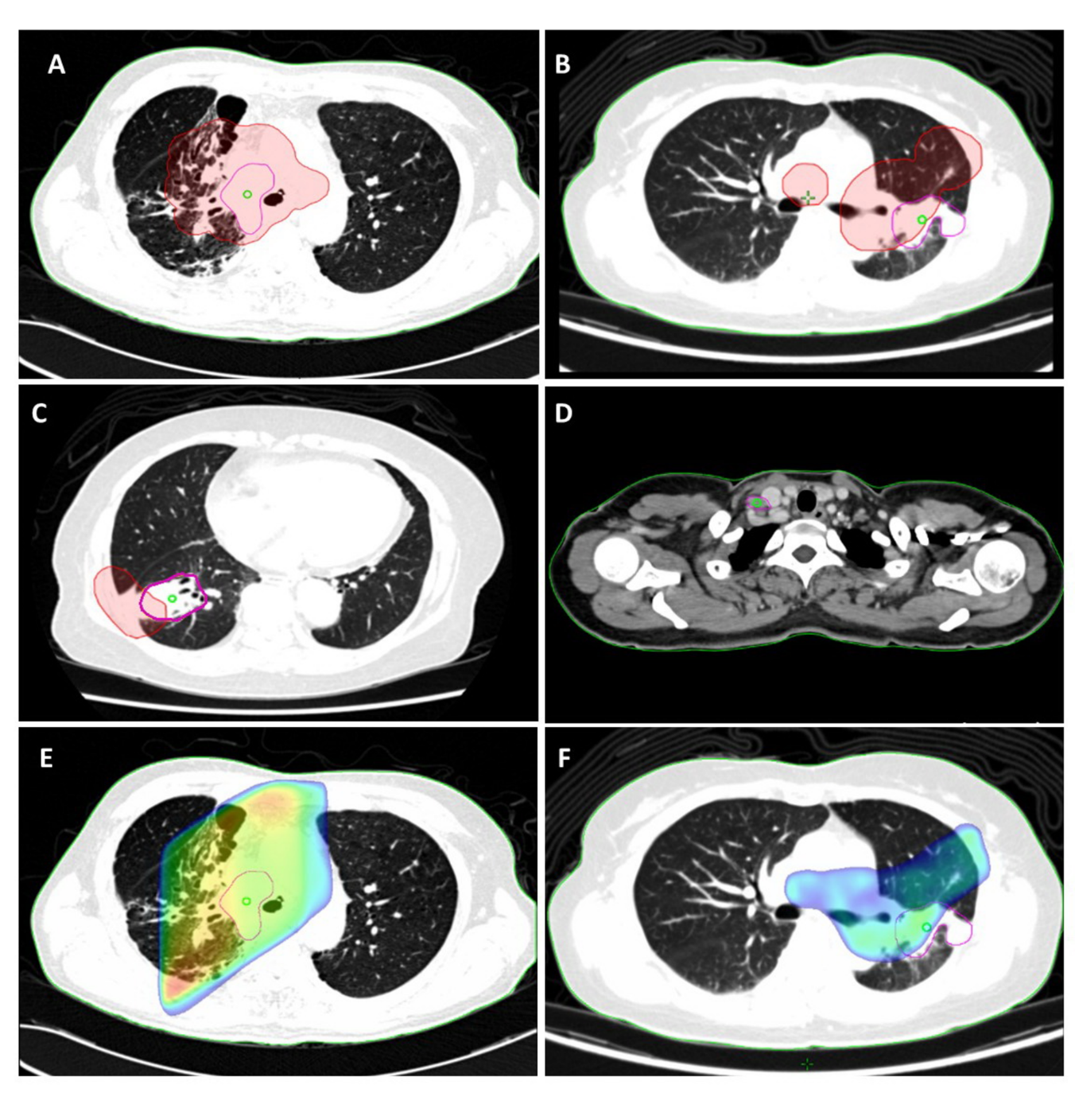

Supplement: Supplementary file 1 — Supplementary Figures. [file 41598_2024_55476_MOESM1_ESM.docx]
